# Dynamics and distribution of paxillin, vinculin, zyxin and VASP depend on focal adhesion location and orientation

**DOI:** 10.1038/s41598-019-46905-2

**Published:** 2019-07-18

**Authors:** Karin Legerstee, Bart Geverts, Johan A. Slotman, Adriaan B. Houtsmuller

**Affiliations:** Erasmus Medical Centre Rotterdam, Department of Pathology, Optical Imaging Centre, Rotterdam, The Netherlands

**Keywords:** Focal adhesion, Cellular imaging

## Abstract

Focal adhesions (FAs) are multiprotein structures that link the intracellular cytoskeleton to the extracellular matrix. They mediate cell adhesion and migration, crucial to many (patho-) physiological processes. We examined in two cell types from different species the binding dynamics of functionally related FA protein pairs: paxillin and vinculin versus zyxin and VASP. In photobleaching experiments ~40% of paxillin and vinculin remained stably associated with a FA for over half an hour. Zyxin and VASP predominantly displayed more transient interactions. We show protein binding dynamics are influenced by FA location and orientation. In FAs located close to the edge of the adherent membrane paxillin, zyxin and VASP were more dynamic and had larger bound fractions. Zyxin and VASP were also more dynamic and had larger bound fractions at FAs perpendicular compared to parallel to this edge. Finally, we developed a photoconversion assay to specifically visualise stably bound proteins within subcellular structures and organelles. This revealed that while paxillin and vinculin are distributed evenly throughout FAs, their stably bound fractions form small clusters within the FA-complex. These clusters are more concentrated for paxillin than for vinculin and are mostly found at the proximal half of the FA where actin also enters.

## Introduction

Focal adhesions (FAs) are the main cellular structures linking the intracellular cytoskeleton to the extracellular matrix (ECM). They are typically several square micrometres in size^1,2^. On the membrane-facing side integrins, transmembrane receptors directly binding to the extracellular matrix (ECM), are the main FA components. A specialised form of actin linked to contractile myosin-II forms the edge of the FA on the cytoplasm-facing side, which we will refer to as F-actin. In between integrins and actin a large and diverse intracellular macromolecular protein assembly is present, with over 200 different reported proteins^3,4^. These include (trans)membrane receptors, other than integrins, adaptor proteins and many different signalling proteins such as kinases, phosphatases and G-protein regulators, which through post-translational modifications add significantly to FA complexity. FAs experience force, the strength of which depends on the combination of myosin-II contractility and the stiffness of the ECM. Because of their importance to the transmission of force from the cell to the ECM and in cell adhesion, FAs are crucial to cell migration. Migration and adhesion are key cellular functions required for many physiological and pathophysiological processes, like embryological development, the functioning of the immune system and also cancer, in particular metastasis^4–6^.

Here we investigated FA location and FA orientation dependent dynamics of four FA proteins, the large scaffold proteins paxillin and vinculin, and two FA proteins that are closely linked to the actin associated with FAs, zyxin and vasodilator-stimulated phosphoprotein (VASP). As adaptor proteins paxillin and vinculin are among the proteins with the most potential binding partners within FAs^3^. In keeping with their having a linking, structural, role they are amongst the first proteins to be recruited to assembling focal adhesion complexes, especially the directly integrin-binding paxillin^7–11^. Vinculin has a head and a tail domain with a flexible linker in between, allowing vinculin to adopt open and closed conformations^12^. Its head domain shares many important binding partners and functions with paxillin, indeed paxillin itself is one of its binding partners^13–16^. However, while Paxillin has no direct interaction with actin, vinculin’s tail domain can directly bind actin filaments as well as the actin-binding proteins α-actinin and the ENA/VASP-proteins^16–20^. Zyxin and VASP are recruited to assembling FAs at much later stages than paxillin or vinculin and are more closely linked to actin^10^. Apart from at FAs zyxin and VASP also cluster at actin-polymerisation complexes, which are periodically distributed along F-actin fibres^21–23^. To stimulate actin polymerisation along FAs zyxin, VASP and vinculin depend on each other for proper functioning^24–29^. Zyxin and VASP, without vinculin, also work together in several other cellular processes such as efficient cell spreading and VASP depends on zyxin for its force-dependent recruitment to FAs^25,30–32^.

Taking advantage of a photoconvertible fluorescent protein in combination with a Fluorescence Recovery After Photobleaching (FRAP) set-up on a confocal microscope, we developed a dedicated assay to specifically reveal the spatial location of the stably bound fraction of a protein. We applied this technique to paxillin and vinculin because FRAP experiments showed both these proteins have strikingly large stably bound fractions of nearly 50%. We visualised within FAs the spatial distribution of the stably bound fractions of paxillin and vinculin, which we found to be concentrated into specific areas along the FA rather than distributed randomly throughout the FA. These concentrated areas of stably bound proteins are most often located in the proximal half of the FA where paxillin is concentrated in small clusters and vinculin is more dispersed.

We also reveal that the binding dynamics of VASP, zyxin, vinculin and paxillin differ with FA location and FA orientation relative to the closest edge of the ventral, or adherent, portion of the plasma membrane. Several factors form gradients based on their distance from the ventral membrane edge, such as actin fibre thickness and connectivity, the concentration of (signalling) molecules and enzyme activity^33–40^, effectively creating different local environments for FAs varying with their distance from the ventral membrane edge. Lastly, by using Monte Carlo based simulations we were able to provide a detailed quantification of the binding dynamics of these four proteins, as well as of the differences seen in FAs with different orientations or cellular locations^41^.

## Results

### FA proteins have stably associated fractions at similar ratios across cell types

**Figure 1:**
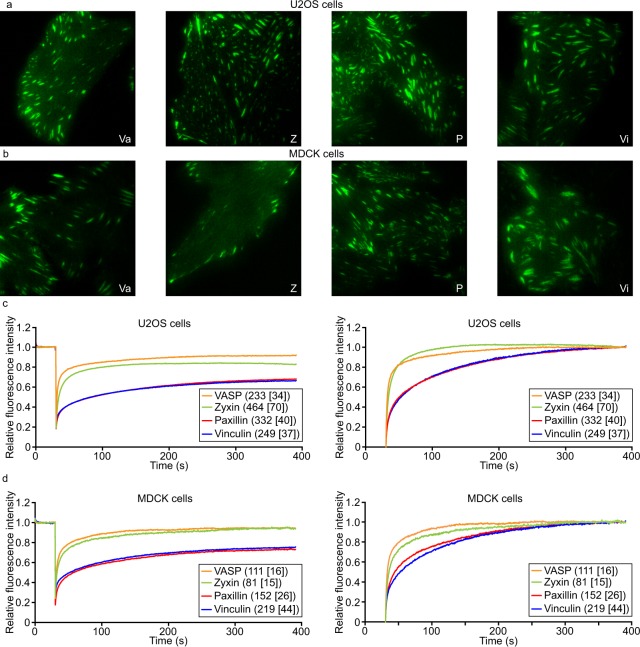
Focal adhesion proteins have stably bound fractions at similar ratios across cell types. (**a**) TIRF images of the four studied proteins expressed in U2OS cells: VASP (Va), zyxin (Z), paxillin (P) and vinculin (Vi). (**b**) TIRF images of the four studied proteins expressed in MDCK cells. (**c**) FRAP-curves for U2OS cells stably expressing GFP-tagged FA proteins. In the left plot fluorescence intensity is expressed relative to prebleach levels, in the right plot relative to immediately postbleach (0) and final recovery levels (1) to facilitate comparison of the recovery rates irrespective of final recovery levels or bleach depths. The numbers between brackets indicate the number of bleached FAs from [number of cells]. (**d**) FRAP-curves for MDCK cells stably expressing GFP-tagged FA proteins.

First, through FRAP-experiments we examined the binding dynamics of fluorescently-labelled paxillin, vinculin, VASP and zyxin at FAs in two different cell-types from two different species; U2OS cells, a human bone cancer cell line, and MDCK dog kidney cells (Fig. 1). Paxillin and vinculin both take about six minutes to reach final recovery levels at ~60% of prebleach fluorescence intensity, whereas both zyxin and VASP recover within two to three minutes to approximately 80 and 90% of prebleach fluorescence intensity. The same pattern of final recovery levels, highest for VASP, intermediate for zyxin and lowest and strikingly similar for vinculin and paxillin, was seen in both U2OS and MDCK cells. To facilitate comparison of recovery rates, irrespective of bleach depth or final recovery levels, recovery curves were expressed relative to intensity immediately after bleaching (0) and final recovery levels (1) (Fig. 1c,d). This highlights the much faster recovery rates of VASP and zyxin versus the highly similar slow recovery rates of paxillin and vinculin. Prolonged FRAP experiments verified paxillin recovery levels remained stable up to 15 minutes post-bleach (Supplementary Fig. S1). To rule out that the incomplete recoveries were due to bleaching of a significant portion of the cytoplasmic protein pool by the intense bleach pulse, we performed experiments where FAs were bleached a second time (Supplementary Fig. S2). If the bleach pulses bleached a significant portion of the protein pool, fluorescence recovery levels after the second bleach pulse would be decreased as a fraction of the bleached proteins would exchange with other bleached proteins. However, after the second bleach pulse fluorescence recovery came to the exact same levels as after the first, demonstrating there is no significant bleaching of the fluorescent protein pool despite the fact that this control experiment contained a second bleach pulse. Instead, the incomplete recovery levels are due to a fraction of the protein pool being stably associated with FAs.

**Figure 2:**
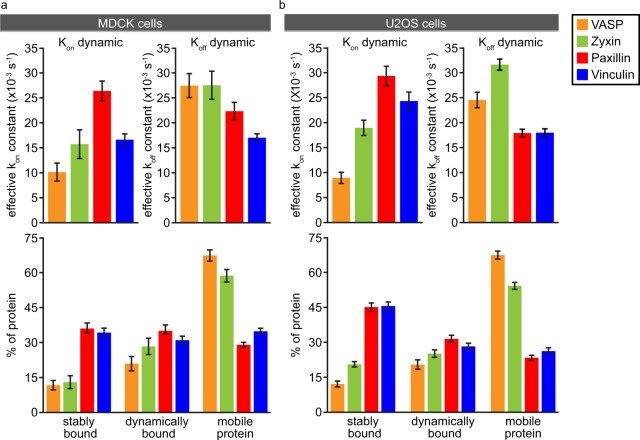
Quantification of the FRAP data. (**a**) Parameters of FA protein dynamics in MDCK cells as determined by fitting the experimental curves shown in Fig. 1 to curves generated by Monte-Carlo based computer simulations. Bar charts of the on- and off-rate constants of the dynamically bound fractions (top panels) and the relative sizes of the stably bound, dynamically bound and mobile fractions (bottom panels). Error bars indicate 2xSEM. VASP n = 111[16], zyxin n = 81[15], paxillin n = 152[26], vinculin n = 219[44] bleached FAs from [cells]. (**b**) Parameters of FA protein dynamics in U2OS cells. VASP n = 233[34], zyxin n = 464[70], paxillin n = 332[40], vinculin = 249[37] bleached FAs from [cells].

We quantified our data by fitting the experimentally-derived FRAP curves to curves generated by Monte Carlo based simulations^41^ (Fig. 2). Briefly, the simulation goes through small time steps. In each step the simulated proteins, which are confined to a volume with the dimensions of a typical cytoplasm, including a nucleus, have chances to step into a random direction, if they are freely diffusing. In addition, they have a chance to get immobilised (effective *k*_*on*_) when close to predefined locations (FAs). Simulated proteins bound to an FA have chances to release (effective *k*_*off*_). Similarly, proteins inside the laser beam during bleaching have a chance to get bleached. The simulation was systematically run with different *k*_*on*_’s and *k*_*off*_’s, leading to the medium and long bound fraction sizes, and their specific residence times. In this way a large database of computer generated FRAP-curves was created from which the one best fitting to the experimental data was selected. The *k*_*on*_’s and *k*_*off*_’s used for the best fitting simulation were used to calculate residence times and fraction sizes^41^ (for details see Materials and Methods). The stably bound fractions obtained in this way for U2OS and [MDCK] cells are (average ± 2*SEM = Standard Error of the Mean): 12.1 ± 1.30, [11.7 ± 2.02]% for VASP, 20.6 ± 1.14, [13.0 ± 2.80]% for zyxin, and as discussed above large and of strikingly similar size for paxillin and vinculin, at 45.1 ± 1.74, [36.0 ± 2.44]% for paxillin and 45.6 ± 1.74, [34.2 ± 1.97]% for vinculin. The simulations also provide accurate estimates of the more dynamically bound fractions: 20.5 ± 2.05, [20.9 ± 3.07]% for VASP, 25.2 ± 1.61%, [28.3 ± 3.51%] for zyxin 31.5 ± 1.54, [35.0 ± 2.51]% for paxillin and 28.2 ± 1.40, [31.0 ± 1.73]% for vinculin. The remainder of the protein pool was associated with FAs so briefly that its residence time was consistent with what would be expected for free diffusion. Hence, we will refer to this fraction as the mobile pool although its proteins may be very briefly immobilised at the FA complex. Additionally, fitting of the data allowed us to determine the average on- and off-rate constants for the dynamically and stably bound fractions, for the dynamic fractions these are plotted. For the stably bound fractions these did not differ significantly between the four proteins (data not shown). The average residence times of the stably bound fractions were over half an hour, which is comparable to previously reported FA lifetimes ranging from approximately 20 to 90 minutes^42–44^. We also examined the lifetime of 100 FAs from 5 cells in time lapse movies, which we found to be 55 ± 6 (2xSEM) minutes. This indicates that a substantial part of the investigated proteins in the stably bound fractions remain associated for the entire lifetime of an FA.

### Examining the spatial location of the stably bound fraction of a protein

**Figure 3:**
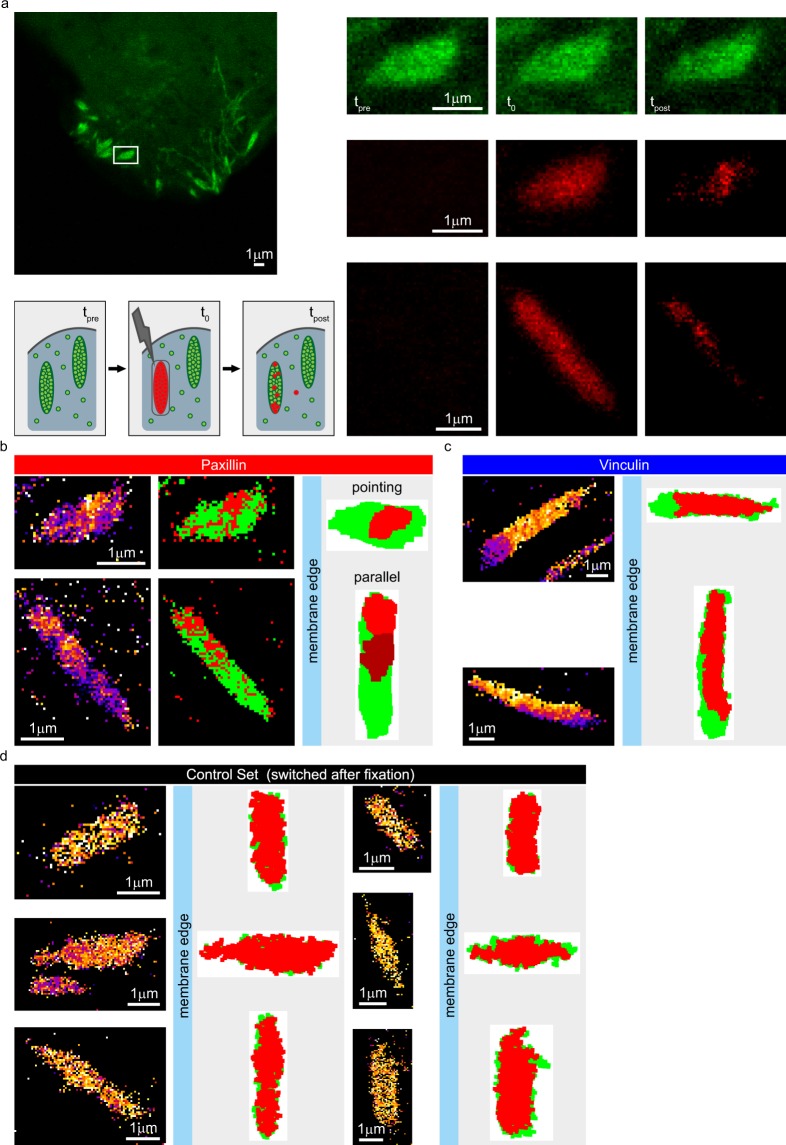
A dedicated assay to specifically visualise the spatial distribution of the stably bound fraction of a protein applied to paxillin and vinculin. (**a**) Cartoon: Schematic overview of a photoconversion experiment. Cells express paxillin or vinculin tagged with the photoconvertible mMaple3. Before the photoconversion all FAs are green (t_pre_). A small region is exposed to a low intensity of 405 nm laser light, converting the mMaple in this area from green to red (t_0_). After 3 minutes, (three times the average residence time of the dynamically bound fraction), another image is taken (t_post_). As the converted volume is small, most mMaple in the cytoplasm is not converted (green), so exchange in the converted FA will almost certainly lead to green proteins coming in. Therefore, at t_post_ the remaining red signal represents the stably associated fraction. Left image: green channel at t_pre_ for a representative pointing FA. Right images: magnification of the green and red channels at each time point for the boxed area, bottom row shows the red channel at each time point for a representative parallel FA. (**b**) Analysis of the representative photoconverted paxillin FAs shown in a. ‘Ratio view’ (RV) images (left), show the ratio of the red signal at t_post_ over the red signal at t_o_. Blue/purple indicates a low ratio, white/yellow a high ratio representing the stably bound fraction. To consistently select the high ratio pixels dynamic thresholds were applied to the RV images (middle image; above threshold pixels red, below threshold green). Stably bound areas were defined as above threshold areas of at least 0.05 µm^2^. Plots were made with the stably bound area(s) in red and the rest of the FA in green (right). To facilitate comparison the distal FA side is always plotted to the left and pointing FAs were plotted with their long axis horizontal while parallel FAs were plotted with their long axis vertical. (**c**) Representative examples of analysed photconversion data for vinculin. Left: RV images; right: rotated plots. (**d**) Representative examples of analysed control data of U2OS cells expressing paxillin-mMaple3 chemically fixed with paraformaldehyde prior to photoconversion.

Before presenting an in-depth analysis of the relationship between FA orientation/location and the dynamic behaviour of the studied proteins, we first present the results of a novel photo conversion assay to specifically investigate the spatial distribution of the observed stably bound fractions of paxillin and vinculin. Since the stably bound fraction of a protein pool cannot be specifically labelled and in FRAP this fraction is bleached, hampering analysis, this required the development of a dedicated assay. Here we used the photoconvertible fluorescent protein mMaple3^45,46^ in combination with a FRAP set-up on a confocal microscope (Fig. 3a). Successful implementation allowed us to specifically visualise the spatial location of the stably associated fractions of either vinculin or paxillin.

The photoconvertible fluorescent protein mMaple3 can be switched from emitting green fluorescence to emitting red fluorescence by exposure to 405 nm laser light at low intensity. In U2OS cells we expressed paxillin or vinculin tagged with mMaple3, which targeted to FAs highlighting them in green fluorescence (Fig. 3a, t_pre_). Using a FRAP set-up on a confocal microscope we briefly exposed a small region of the cell, tightly enclosing one or two FAs, to 405 nm laser light. This switched the mMaple-tagged paxillin molecules, only within the exposed area, from emitting green fluorescence to emitting red fluorescence. Thus, the FA(s) within the exposed area emitted red fluorescence when excited at the appropriate wavelength (Fig. 3a, t_0_). Next, we waited for three minutes, since this is ~3 times the average dynamic residence time for paxillin/vinculin, the vast majority of the dynamically binding proteins exchange. As only a small portion of the cell was briefly (in the hundreds of milliseconds range) exposed to the 405 nm laser light the unconverted green mMaple-tagged protein is present in vast excess compared to the converted red mMaple-tagged protein. Therefore, exchanging converted protein will almost certainly be replaced by unconverted protein, whereas stably associated converted proteins remain in the FA, revealing the spatial distribution of the stably and dynamically bound fractions of the studied proteins (Fig. 3a, t_post_).

To improve visualisation of stably bound fractions within the context of entire FAs we made ‘ratio view’ (RV) images (Fig. 3b–d, left images). In these images a colour-coded scale is used to show on a pixel by pixel basis the ratio of the photoconverted signal still present at t_post_ over the converted signal present at t_0_. Blue/purple pixels in the RV image indicate a low ratio of the converted signal after 3 minutes, visualising the dynamically bound fraction. White/yellow in the RV image means a high ratio of the converted signal at t_post_, visualising the stably bound fraction.

To consistently differentiate between dynamically and stably bound areas, we applied a threshold to the RV images (Fig. 3b, middle images). To see if the stably bound fraction is spread evenly and randomly over the FA or is instead concentrated into specific areas, we examined above threshold areas of 0.05 µm^2^ or larger. We created plots wherein these stably bound area(s) are plotted in (shades of) red and the rest of the FA in green (Fig. 3b–d, right images). To allow for easy comparison between FAs, for each FA we determined its distal side, the side which lies closest to the ventral membrane edge, then rotated the FA so that this side is always plotted to the left. FAs with their long axis more or less perpendicular to the ventral membrane edge, will be referred to as ‘pointing FAs’ and are plotted with their long axis horizontally, FAs with their long axis roughly parallel to this membrane edge are referred to as ‘parallel FAs’ and are plotted with this axis vertically (for a precise definition see below and Materials and Methods).

In addition, we performed control experiments where U2OS cells expressing paxillin-mMaple3 were chemically fixed prior to the photoconversion experiments (Fig. 3d). For these controls ratios were high throughout the FA in the RV images and the stably bound areas covered nearly the entire FA in the plots, as expected in fixed cells where proteins cannot exchange.

**Figure 4:**
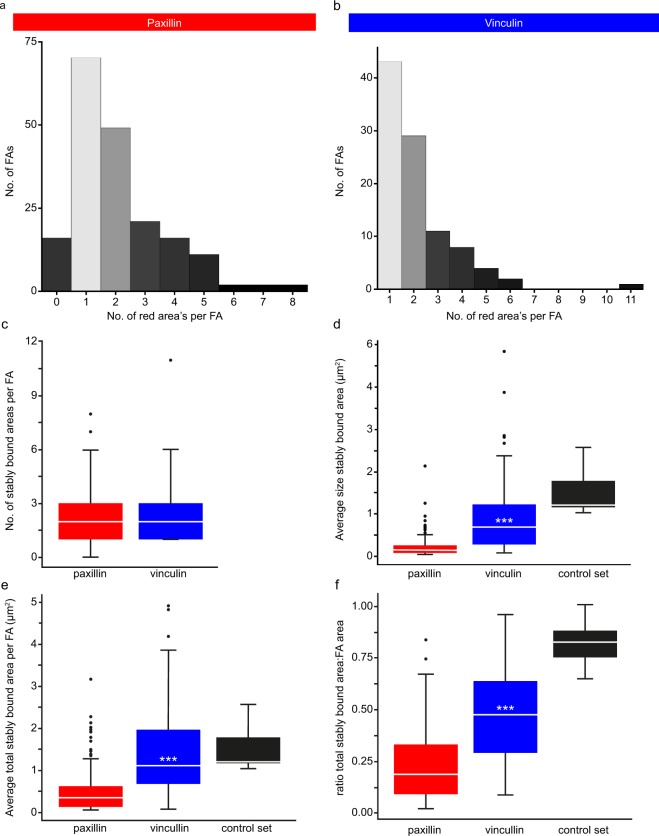
Quantification of the photoconversion experiments. (**a**,**b**) Histograms of the number of stably bound areas (above threshold areas larger than 0.05µm^2^ after application of a dynamic threshold on the RV image) per FA for U2OS cells expressing paxillin-mMaple3 (a, n = 189 FAs in 153 cells) or vinculin-mMaple3 (b, n = 98 FAs in 84 cells). Note that for both proteins the largest number of FAs has one stably bound area. (**c**) Tukey style boxplots of the data shown in A. White lines: median; asterisks: significant p-values generated by two sided Mann Whitney tests: *p < 0.05, **p < 0.01, ***p < 0.001. (**d**) Tukey style boxplots of the surface area (µm^2^) of the individual stably bound areas for all FAs with stably bound areas (paxillin n = 173, vinculin n = 98, control n = 7). The control set contains cells expressing paxillin-mMaple3 which were chemically fixed prior to the photoconversion experiment. White lines and asterisks as in c. (**e**) Tukey style boxplots of the average total surface area (µm^2^) covered by stably bound areas per FA for all FAs (for n see d). White lines and asterisks as in c. (**f**) Tukey style boxplots of the ratio of the total area of the FA covered by stably bound area(s) over the total FA surface area (for n see **d**). White lines and asterisks as in c. Note that the proportion of the FA covered by stably bound area(s) is significantly larger for vinculin than paxillin, indicating that paxillin is more concentrated than vinculin since FRAP experiments have shown paxillin and vinculin have nearly identically sized stably bound fractions.

From the representative examples it seems that for the vinculin FAs the proportion of the FAs covered by the stably bound fraction was much larger than for the paxillin FAs. This was confirmed by a quantitative analysis of the stably bound areas for paxillin (n = 189 photoconverted FAs from 153 cells) and vinculin (n = 98 FAs from 84 cells). The number of stably bound areas per photoconverted FA did not differ significantly between paxillin and vinculin, for both proteins the median value was two although the most common number of stably bound areas per FA was one (Fig. 4a–c). However, the size of individual stably bound areas was more than 4 times smaller for paxillin than for vinculin, medians 0.14 and 0.69 µm^2^ respectively (Fig. 4d). The summed stably bound area per FA, irrespective of the number of stably bound spots this area is spread over in the FA, was also more than three times smaller for paxillin than for vinculin (Fig. 4e). To determine the proportion of FAs covered by stably bound areas we divided the summed stably bound surface area per FA by the total surface area of that FA, making this value independent of FA size. For paxillin this value corresponded to less than one fifth of the FA (median 0.19), while for vinculin almost half (median 0.48) of the FA was covered by stably bound areas. Since the FRAP data showed the size of the stably bound fraction is nearly identical for paxillin and vinculin, this indicates that stably bound paxillin proteins are more concentrated at FAs than stably bound vinculin proteins.

**Figure 5:**
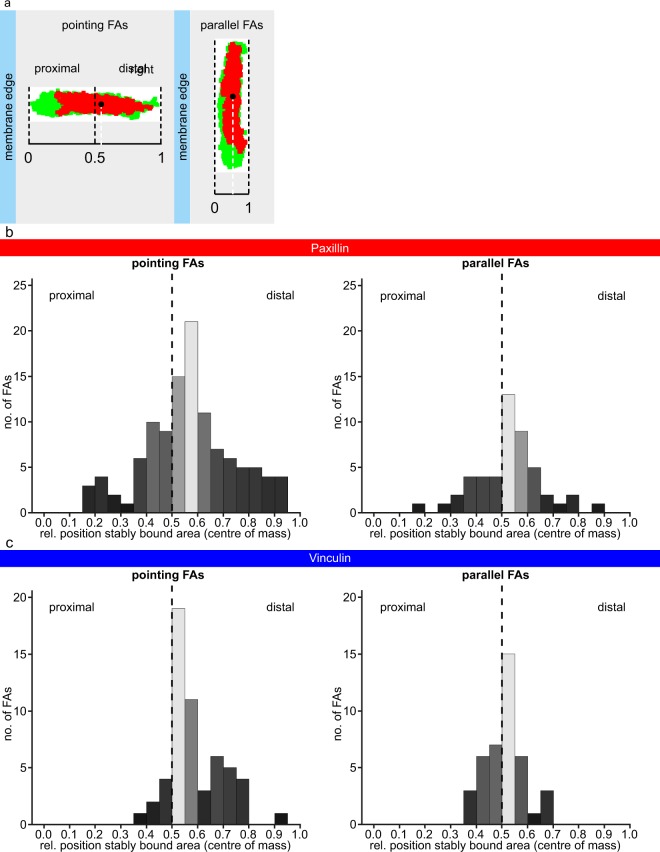
The stably bound paxillin and vinculin are concentrated more often in the proximal half of FAs. (**a**) Analysis of the location of the stably bound fraction of an FA relative to the closest ventral membrane edge. In the plots FAs are plotted with their distal side, which in the cells lies closest to the edge of the ventral membrane, on the left. The centre of mass of the stably bound area(s) is determined relative to the normalised length of the major axis of the FA for pointing FAs, or of the minor axis for parallel FAs. (**b**) Histogram of the centres of mass of the stably bound area(s) relative to the closest edge of the ventral membrane for all pointing FAs (left, n = 113)/parallel FAs (right, n = 49) that have stably bound areas for cells expressing paxillin-mMaple3. (**c**) Histogram of the centres of mass of the stably bound area(s) relative to the closest edge of the ventral membrane for all pointing FAs (left, n = 56)/parallel FAs (right, n = 41) that have stably bound areas for cells expressing vinculin-mMaple3.

Additionally, for each photoconverted FA we calculated the average weighted (by area) gravitational centre for the stably bound area(s), which we expressed relative to the major axis of the FA in the case of pointing FAs, or the minor axis of the FA for parallel FAs (Fig. 5a). This allows comparison of the location of the stably bound areas among all FAs irrespective of FA length. The stably bound fraction was located at the proximal FA half for 69% of all converted paxillin FAs and for 75% of all converted vinculin FAs (Fig. 5b,c), which is the FA end where the F-actin fibre also enters the FA (Supplementary Fig. S3).

Careful examination shows not all mMaple3 in the FAs is converted from green to red since, apart from in the red channel, the FA was also still clearly visible in the green channel immediately after photoconversion (Fig. 3a, t_0_), which was true for all converted FAs. This means that for the green signal seen at t_post_ we do not know if it comes from exchanged proteins or from stably bound proteins that were not converted at t_0_. However, the entire FA is clearly visible in the red channel at t_0_, indicating a reasonable proportion of the tagged protein was converted throughout the FA, also true for all FAs. For this reason, even though we are clearly not photoconverting the whole population of tagged protein present in the FA, we are confident that the converted proportion of the tagged protein represents a fair and random sample taken from the entire population of tagged proteins associated with the FA, as is also shown by the control experiments on fixed samples.

### Categorisation of focal adhesions based on their position and orientation

Several factors form gradients based on their distance from the edge of the ventral membrane, such as actin fibre thickness and connectivity, the concentration of (signalling) molecules and enzyme activity^33–40^. Such gradients effectively create different local environments for FAs varying with their distance from the ventral membrane edge. To investigate whether such variation in local environments influences FA protein dynamics, we further subdivided the FRAP data shown globally in Fig. 1.

**Figure 6:**
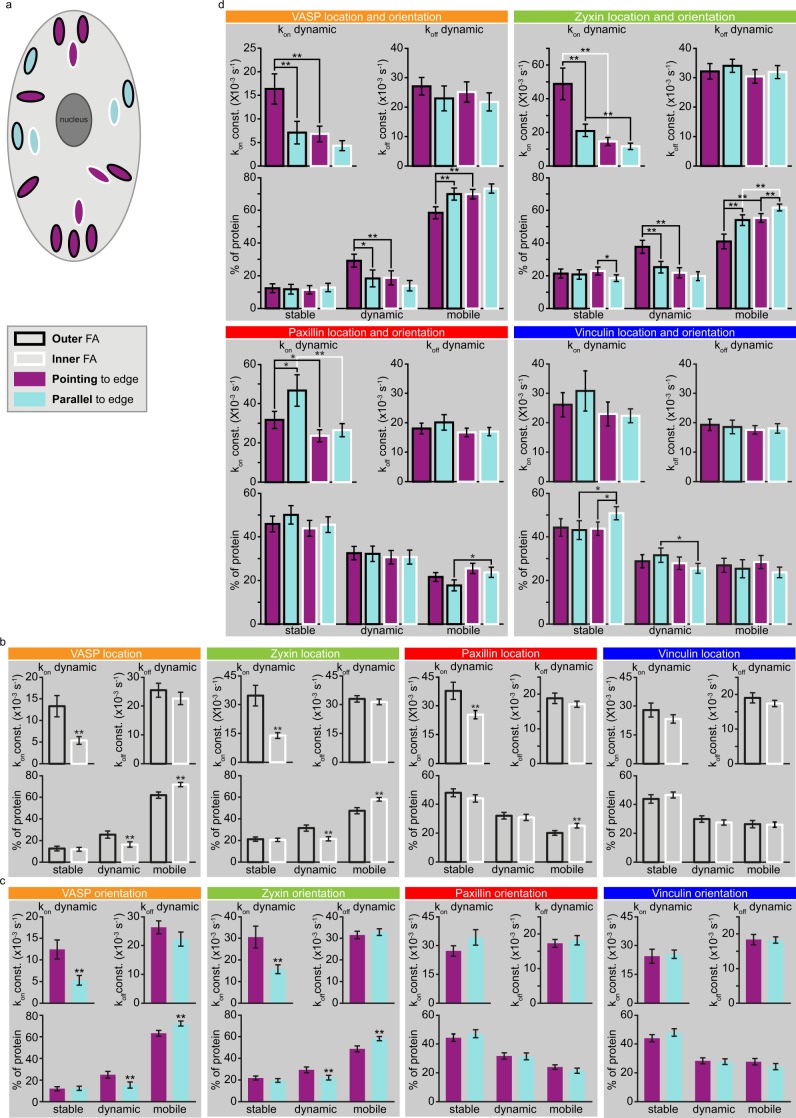
FA distance from and/or orientation relative to the closest edge of the ventral membrane influence its dynamics. (**a**) Cartoon illustrating classification of FAs based on distance from or orientation relative to the closest edge of the ventral (adherent) portion of the plasma membrane. ‘Outer’ FAs (black outline) are close to the edge of the ventral membrane, ‘inner’ FAs (white outline) are located away from the edge with outer FAs located in between. In ‘pointing’ FAs (purple) the longest axis is oriented more or less perpendicular to the closest membrane edge, and in ‘parallel’ FAs (blue) the longest axis is more or less parallel to the edge. (**b**) Quantification of U2OS cell FRAP data based on FA distance from the closest edge of the ventral membrane. VASP[34]: outer n = 100, inner n = 114, Zyxin[70]: outer n = 185, inner n = 260, Paxillin[40]: outer n = 120, inner n = 200, Vinculin[37]: outer n = 91 inner n = 158 FAs from [cells]. Error bars indicate 2xSEM. Asterisks indicate significant p-values generated by two sided Mann Whitney test: *p < 0.01, **p < 0.001. (**c**) Quantification of U2OS cell FRAP data based on FA orientation relative to the closest edge of the ventral membrane. VASP[34]: pointing n = 109, parallel n = 93, Zyxin[70]: pointing n = 199, parallel n = 209, Paxillin[40]: pointing n = 136, parallel n = 134, Vinculin[37]: pointing n = 119, parallel n = 95 FAs from [cells]. Error bars and asterisks as above. (**d**) Parameters obtained by fitting the FRAP data after categorising the FAs based on the combination of their distance from and their orientation relative to the closest edge of the ventral membrane. This is especially revealing for VASP and zyxin, as it shows that it are specifically the outer and pointing FAs that have strongly altered VASP and zyxin dynamics compared to all other FA types. N-numbers per protein for consecutively: outer and pointing, outer and parallel, inner and pointing and inner and parallel FAs: VASP: 64, 34, 45, 58, Zyxin: 90, 90, 104, 118, Paxillin: 65, 51, 67, 82, Vinculin: 56, 35, 63, 60. Error bars and asterisks as above.

First, all FAs were grouped on the basis of their distance from the closest ventral membrane edge. ‘Outer’ FAs are located close to this edge, ‘inner’ FAs are positioned further inwards with outer FAs located between them and the closest adherent membrane edge (Fig. 6a,b).

In addition, we noticed that FAs are mostly orientated with their long axis either roughly perpendicular or roughly parallel to the closest adherent membrane edge. FAs were classified as ‘pointing’ when the angle between their long axis and the closest ventral membrane edge was 90° ± 30°. FAs were classified as ‘parallel’ when the angle between their long axis and the closest ventral membrane edge was 180° ± 30° (Fig. 6a,c). Of the 1278 bleached FAs (see Materials and methods for a specification per examined protein) 184 fell outside these criteria, which is 14%, whereas one third (60°/180°) would be expected if FA orientation would be random.

Finally, the adhesions were grouped based on the combination of these two criteria which results in four groups: A outer and pointing, B outer and parallel, C inner and pointing and D inner and parallel FAs (Fig. 6a,d).

### Differences in FA protein dynamics based on FA location

Zyxin and VASP exchange dynamics followed the same trend when comparing outer to inner FAs (Fig. 6b). At outer FAs the on-rate constant of the dynamically associated fraction was more than twice as large, leading to a significantly increased dynamically bound fraction and a significantly decreased mobile pool.

Similarly, for paxillin the on-rate constant of the dynamically associated fraction was significantly increased at the outer FAs while the mobile pool was significantly decreased. Unlike for zyxin/VASP this last was not caused by a significant increase of the dynamically associated fraction specifically, but rather by the sum of individually insignificant increases of the dynamically and the stably associated fractions.

### Differences in FA protein dynamics based on FA orientation

Zyxin and VASP also showed similar trends in their dynamic behaviour when comparing parallel to pointing FAs (Fig. 6c). At pointing FAs the on-rate constant of the dynamically associated fraction is more than twice as large, resulting in a significantly increased dynamically bound fraction and a significantly decreased mobile pool.

For paxillin and vinculin none of the measured parameters were significantly altered in pointing FAs compared to parallel FAs.

### Differences in FA protein dynamics based on both FA location and orientation

Having found that FA location and orientation separately correlate to the dynamics of FA associated proteins, we next examined the four possible combinations of FA location and orientation.

Again, the dynamics of zyxin and VASP followed a similar trend, with their dynamics at the outer and pointing FAs clearly standing out from their dynamics at any other FA type (Fig. 6d). Specifically, compared to either outer and parallel or to inner and pointing FAs the size and the on-rate constants of the dynamically associated fraction was significantly increased and the mobile pool was significantly decreased. For zyxin additional significant differences in dynamic behaviour were observed when comparing the remaining FA types, however these were all of a much smaller magnitude than those seen when comparing zyxin/VASP dynamics at outer and pointing FAs to zyxin/VASP dynamics at any other FA type.

For paxillin significant differences were also observed when comparing the four different FA types, but unlike for zyxin/VASP no single FA type clearly stands out from the rest. At the outer and pointing FAs the on-rate constant of the dynamically associated fraction is decreased compared to at the outer and parallel FAs, but increased compared to at the inner and pointing FAs. At the outer and parallel FAs the on-rate of the dynamically associated fraction is increased and the mobile pool decreased compared to at the inner and parallel FAs.

Similar to the lack of correlation between vinculin dynamic behaviour and FA location or orientation separately, the combination of FA location and orientation also had little effect, but there were some significant differences. At the inner and parallel FAs the stably bound fraction was increased compared to at either the outer and parallel or the inner and pointing FAs while the dynamically associated fraction was significantly decreased compared to at the outer and parallel FAs.

## Discussion

Here we studied the dynamics of two pairs of functionally related focal adhesion proteins, paxillin/vinculin and zyxin/VASP, in two different, slow moving, non-fibroblast cell types on a collagen coating. The quantitative data were highly consistent between the cell types from different species, suggesting these findings are not cell-type specific and are relevant for FA function.

**Figure 7:**
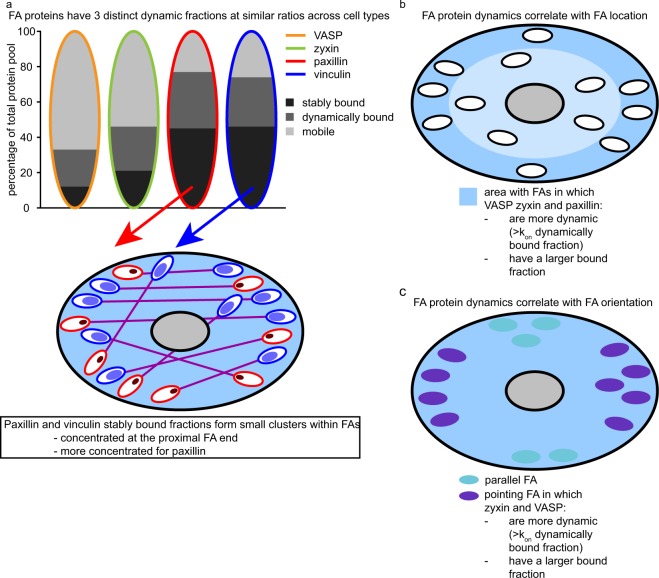
Schematic overview of the data. (**a**) Model showing the ratio between the 3 dynamic fractions, stably bound, dynamically bound and mobile protein for each of the studied proteins as revealed by fitting of the experimental FRAP curves to curves generated by computer-based simulations (top panel). The bottom panel schematically shows the location of stably bound paxillin and vinculin within FAs as revealed by the photoconversion assay. Stably bound paxillin and vinculin form small clusters that are most often located in at the proximal FA end, for paxillin these cluster are more concentrated than for vinculin. (**b**) Schematic overview of how FA protein dynamics correlate with FA location, VASP, zyxin and paxillin are more dynamic (have a higher on-rate constant for the dynamically bound fraction) and a larger stably bound fraction at FAs located close to the ventral membrane edge. (**c**) schematic overview of how FA protein dynamics correlate with FA orientation, VASP and zyxin are more dynamic (have a higher on-rate constant for the dynamically bound fraction) and a larger bound fraction at FAs orientated with their long axis more or less perpendicular to the ventral membrane edge.

We present a novel assay to specifically visualise the spatial distribution of stably associated proteins. A strong advantage of this new technique, over for example bleaching a single FA and following its recovery, is that we directly visualise the stably bound fraction. Moreover, the entire FA remains visible as converted proteins in the dynamic and mobile pools exchange with unconverted. We successfully applied this assay to paxillin and vinculin in living cells. This revealed that stably bound paxillin and vinculin are accumulated in small clusters. Paxillin clusters are smaller and more concentrated than vinculin clusters (Figs 4d and 7a), since FRAP experiments showed that both have similar stably bound fractions. This may be because paxillin directly binds to integrins, which during the early phases of FA complex formation strongly cluster together. A recent study found this clustering to be even more pronounced than previously thought, with active and inactive integrins forming discrete nanoclusters^47^. Conversely, vinculin directly binds to force-bearing F-actin potentially pulling the vinculin clusters further apart. Within this context it is interesting that for pointing FAs the paxillin and vinculin small clusters are mostly located at the proximal half of the FA, the side of the FA where the F-actin stress fibre also enters. This ties in well with a previous study showing inactive vinculin is enriched at the FA tip closest to the edge of the ventral membrane, while active vinculin is enriched at the proximal tip, since activated vinculin is known to be more stably associated^48,49^. In any case we thus show that while paxillin and vinculin proteins are evenly distributed along the FA, their stably bound fractions form small clusters within the FA-complex, most frequently at its proximal end.

A large number of previous studies have examined the dynamics of paxillin, vinculin, zyxin or VASP in fibroblasts^11,48,50–63^, but never together in the same study. Moreover, each study used different culturing and quantitative analysis methods, leading to considerable differences in reported quantitative parameters. For instance, for paxillin, half times to full recovery were between 1.5 and 41 seconds, the times until final recovery between 30 and 200 seconds, and mobile fractions between 60% and nearly 100%. The period fibroblasts were cultured on fibronectin prior to imaging ranged from 15 minutes to 48 hours which influences spreading level, a factor known to affect FAs including maturation, which has been shown to inhibit vinculin dynamics^54^. Moreover, different culturing conditions may also affect FA composition and protein phosphorylation status, conformational states and this may influence protein dynamics^11,50,52–54,56,58,62^. The net result of this is a large variation in the reported quantitative parameters for these FA proteins, hampering comparison with the parameters presented here, as well as the parameters for the different proteins examined in different studies. However, it may still be noted that the speed of paxillin and vinculin recovery we observed was considerably slower than in previous studies, and we found larger stably associated fractions for these proteins. Both parameters were consistent between our two different cell types, which were from different species, suggesting they are not cell-type specific and are relevant for FA function. Yet neither of the two cell types used in this study show as much or as fast unstimulated migration as fibroblasts. Thus, the slow recovery and large immobile fractions we observed might be typical of cells displaying less or slower unstimulated migration.

We demonstrated that for each of the studied proteins three different dynamic pools are present in FAs: 1) a pool with stable associations ( > 30 minutes), 2) a dynamically exchanging pool with shorter interactions (~1 minute) and 3) a very dynamic pool with interactions so brief that they could not be distinguished from free diffusion (referred to as the mobile pool). In both cell types the stable fraction is small for VASP, somewhat larger for zyxin and largest and surprisingly similar for paxillin and vinculin (Fig. 7a). Consistently, previous studies measuring the recovery of both paxillin and vinculin also reported highly similar immobile fractions^55–58^, but very different amongst studies. Since FAs show a sliding type of movement, both in migrating and in stationary cells, they are typically regarded as highly dynamic complexes^64,65^. However, the average residence time of all stably associated fractions was relatively long compared to previously reported FA lifetimes and the average FA lifetime of 55 ± 6 minutes observed here^42–44^, indicating the proteins in these fractions remain associated for a large part of the lifetime of a FA and revealing some properties of FAs are less dynamic than previously thought.

Furthermore, we show that protein binding dynamics differ with FA orientation and location relative to the closest edge of the ventral membrane (Fig. 7b,c). This was especially true for zyxin and VASP, for which the on-rate constant of the dynamically associated fraction was more than twice as high at outer vs inner FAs and the same was true for pointing vs parallel FAs. Further investigation revealed dynamic on-rate constants were specifically increased at FAs that are outer as well as pointing. Increased on-rate constants can be the result of either increased numbers of available binding sites or of increased affinity for these sites. Because the off-rate constants of the dynamic fractions were not significantly altered, the increased on-rate constants lead to significantly increased sizes of the dynamically bound zyxin/VASP fractions at these FAs. A likely function of which is to facilitate the coupling of actin by increasing the number of available actin binding sites, as both proteins directly bind actin. Strong links between actin and FAs that lie close and perpendicular to the edge of the ventral membrane (i.e. are outer and pointing) are presumably needed to generate the force required to protrude or retract the ventral membrane. The effects of FA location and orientation on paxillin and vinculin dynamics are more subtle, for vinculin only the combination significantly correlated with its dynamics. For paxillin at outer FAs the on-rate constant of the dynamically associated fraction was significantly increased and the mobile pool significantly decreased, meaning an increased total (stable and dynamic) bound fraction. In spite of the increased on-rate constant, the dynamic fraction was not increased. This is due to the large decrease of the mobile pool, decreasing the number of paxillin molecules available for binding. With respect to biological function the increased total bound fraction of paxillin at outer FAs may be needed to deal with the increased force created by increased bound fractions of zyxin/VASP. Furthermore, an increase in the on-rate constant of the dynamically bound fraction of a structural component like paxillin, may stimulate the dynamics of the entire FA. This is supported by the increased on-rate constants of the dynamically bound VASP/zyxin fractions at outer FAs. It could be advantageous for FAs at the edge of the ventral membrane to be more dynamic when a cell is exploring its immediate environment by protruding its membrane at different areas, but is not yet committed to moving in a particular direction.

Overall it is remarkable how similar the dynamics of vinculin/paxillin are, and different from the also similar dynamics of zyxin/VASP, this last particularly with respect to the impact of FA orientation and location. The vinculin head domain and paxillin as well as zyxin/VASP share many functions and binding partners, including each other, potentially contributing to the similar binding dynamics. For the vinculin head domain and paxillin the most notable shared binding partner is talin, while zyxin/VASP both bind actin and work together in several cellular processes such as efficient cell spreading^13–16,25,30–32^. However vinculins tail domain shares many functions and binding partners with zyxin/VASP, including zyxin/VASP themselves which depend on vinculin for many of the actin regulating processes they are involved in^16–20,24–29^. The remarkably similar vinculin/paxillin dynamics suggest that vinculins head domain influences its dynamics more strongly than its tail domain. This is perhaps because vinculin, like paxillin, enters a newly forming FA complex when many of the proteins interacting with its head domain are already associated, while most of the interaction partners for its tail domain are not yet present. When at later stages the latter enter the FA complex, a large proportion of the vinculin molecules will already be extensively involved in interactions through their head domains. Later additional interactions through their tail domains will have little influence on their binding dynamics. Interestingly, in other studies looking at different aspects of protein behaviour at FAs, a split between paxillin/vinculin versus zyxin/VASP was also observed. For instance, zyxin/VASP dissociate from disassembling FAs earlier than paxillin/vinculin, including in response to actomyosin-II inhibition^21,58^. When stress fibres thicken, in response to mechanical stress or to the actin stabilizer jasplakinolide, VASP/zyxin rapidly translocate from FAs to the thickening stress fibres while vinculin/paxillin remain associated^28,66^. Zyxin is completely lost from FAs in response to actin polymerisation inhibition, while vinculin levels remain unchanged^52^. Overexpression of the zyxin LIM-domain causes the loss of endogenous zyxin and VASP from FAs while vinculin levels remain unchanged^27,67^. Thus, at FAs a distinction between paxillin/vinculin and VASP/zyxin behaviour seems to be a previously unrecognised recurring theme.

In summary, we examined the dynamics of two pairs of functionally related FA proteins, paxillin/vinculin and zyxin/VASP. For each protein we demonstrated the presence of a stably associating, a dynamically exchanging and a mobile pool within FAs. Furthermore, we show that protein binding dynamics differ with FA orientation and location. This is especially true for zyxin and VASP and most especially at FAs that are located close to the nearest ventral membrane edge and orientated with their long axis perpendicular to it. At these FAs there are significantly more zyxin and VASP proteins binding in a significantly more dynamic manner, potentially to facilitate the coupling of actin to these FAs since both proteins are directly actin-binding. The effects of FA location and orientation on paxillin and vinculin dynamics are more subtle, but it is noteworthy that at FAs close to the membrane edge paxillin binding is significantly more dynamic, presumably stimulating the dynamics of these FAs since paxillin is a key structural component. We noted there is a distinction between the dynamics of paxillin/vinculin and VASP/zyxin, which a literature search revealed is a previously unrecognized but recurring theme in their behaviour at FAs. Finally, we presented and applied a novel assay to specifically visualise the spatial distribution of stably associated proteins in living cells. We showed that while paxillin and vinculin proteins are distributed evenly throughout FAs, their stably bound fractions form small clusters within the FA-complex. These clusters are most frequently found at the proximal FA end, the FA end where the F-actin stress fibre also enters and are significantly more concentrated for paxillin than for vinculin.

## Materials and Methods

### Cell culture

MDCK cells were cultured in DMEM (Lonza) and U2OS cells in phenol-red free DMEM (Lonza) at 37 °C and 5% CO_2_. Culture media were supplemented with 10% FCS (Gibco), 2mM L-glutamine (Lonza), 100 U/ml Penicillin and 100 µg/ml Streptomycine (Lonza) and to maintain stable cell lines with 100 mg/ml G418. Transfections were performed using Fugene (Promega), followed by selection with G418 when creating stable cell lines. For experiments 24 mm round glass coverslips were coated overnight at 4 °C with PureCol bovine collagen type I (Advanced Biomatrix) at a final concentration of 10 µg/ml. Cells were plated onto coated coverslips 24–48 h prior to imaging, for photoconversion experiments the medium was replaced ~1 h prior to imaging with FluoroBrite DMEM (Thermo Fisher Scientific) to minimize autofluorescence.

### Constructs

The zyxin-GFP plasmid was created by replacing the mMaple3 in Zyxin-mMaple3 (Addgene101151) with eGFP from eGFP N1 (Clontech) as a BamHI NotI fragment.

The VASP-GFP, vinculin-GFP, paxillin-GFP and paxillin-mMaple3 plasmids were based on the VASP-mTurquoise (Addgene 55585), Vinculin-mTurquoise (Addgene 55587) and paxillin-mTurquoise (Addgene 55573) vectors, respectively. These use the multiple cloning site as a linker region between the protein and mTurquoise, hampering a simple colour swap. To still allow sticky-end ligation the protein, fluorescent label and empty vector backbone were all separately isolated as restriction fragments. These were ligated using sticky ends in 2 steps: (1) the protein to the fluorescent label (2) the created insert into the vector backbone. This strategy was applied to create the following constructs:

VASP-GFP: The vector backbone was isolated from paxillin-mTurquoise as an AgeI NotI fragment, VASP from VASP-mTurquoise as an AgeI BamHI fragment and GFP from eGFP N1 as a BamH1 Not1 fragment.

Vinculin-GFP: The vector backbone was isolated from paxillin-mTurquoise as a NheI NotI fragment, vinculin from vinculin-mTurquoise as a NheI EcoRI fragment and GFP from eGFP as an EcoRI NotI fragment.

Paxillin-GFP: The vector backbone was isolated from paxillin-mTurquoise as a BamHI NotI fragment, paxillin from paxillin-mTurquoise as a BamHI HindIII fragment and GFP from eGFP as a HindIII NotI fragment.

To create paxillin-mMaple3 the vector backbone was isolated from paxillin-mTurquoise as a BamHI NotI fragment and paxillin as a HindIII BamHI fragment. The mMaple3 was isolated from zyxin-mMaple3 (Addgene 101151) through PCR with primers GCAGAACCATCTCCCACAATGAC (FW) and GTTGCCCTCCATCCTCAGTTTG (RV), the mMaple was isolated from the PCR product as an HindIII NotI product. The paxillin was ligated to the mMaple3 which was then inserted into the vector backbone.

To create vinculin-mMaple3 the GFP in Vinculin-GFP was replaced with mMaple3 from paxillin-mMaple3 as an EcoRI NotI fragment.

All constructs were checked through sequencing, one silent mutation was found (paxillin-GFP bp1461 C to G).

### Actin staining

U2OS cells stably expressing paxillin-GFP were stained with phalloidin-CF405 (Biotium) according to the manufacturer’s protocol and dehydrated with ethanol. Imaging was done on a Zeiss Elyra PS1 system equipped with a LSM 780 confocal unit set to confocal mode and using a 63× 1.4NA oil immersion objective. For the green/cyan channel a 488 nm/405 nm laser was used for excitation and emission filters were set to 495–600 nm/410–480 nm respectively, frame averaging was set to 4. Images were collected as 16 bit with 512 by 512 pixels, z-stacks of 5 slices with a distance of 300 µm were recorded.

### Time lapse TIRF imaging

Time lapse movies were made for 18 hours at 10 minute intervals on a Nikon Ti-Eclipse inverted microscope equipped with a TIRF unit and a 16 bit EM CCD camera (Photometrics) in TIRF mode using a 60× 1.45NA oil immersion objective (Apochromat TIRF). Cells were maintained at 37 °C and 5% CO_2_ using a stage-top incubator (Tokai Hit). These were used to determine the lifetimes of 100 FAs from 5 different cells.

### FRAP experiments

#### Live-cell imaging

All FRAP data was acquired on a Nikon Ti-Eclipse inverted microscope equipped with a TIRF unit, a 3D FRAP scanning unit (Roper) and a 16 bit EM CCD camera (Photometrics) in TIRF mode and using a 60× 1.45NA oil immersion objective (Apochromat TIRF). Cells were maintained at 37 °C and 5% CO_2_ using a stage-top incubator (Tokai Hit). Images were taken for 30 s prebleach and 6 minutes postbleach at 500 ms intervals. The FRAP unit allowed the efficient bleaching of 2 by 2 µm squares ~ 15–25 FAs spread over the field of view, which contained (portions of) several different cells, well within 300 ms. For U2OS cells the number of bleached FAs from [number of cells] were for VASP 233[34], zyxin 464[70], paxillin 332[40], vinculin 249[37] and for MDCK cells for VASP 111[16], zyxin 81[15], paxillin 152[26] and vinculin 219[44].

#### Data analyses

In ImageJ software^68^ extended in the FIJI framework^69^ ROIs were manually drawn around efficiently bleached (portions) of FAs, as well as a few unbleached FAs for control purposes and empty areas for background measurement. To control for monitor bleaching and/or bleaching of too high a proportion of the entire protein pool any experiments where the average intensity of the unbleached FAs fell below 90% of original levels were discarded. Separate experiments on MDCK cells expressing Paxillin-GFP, where the same FA was bleached a second time 6 minutes after the original bleach pulse and followed a further 6 minutes ruled out that a significant fraction of the fluorescent protein pool was bleached using our experimental setup (Fig. S2). Data from any FAs not bleached to < 20% of their average prebleach levels was excluded from analysis, as were any FAs that were not in a stable state. The resulting fluorescence intensity data was background-corrected and normalised to prebleach levels using the following formula:

$${I}_{norm}=\frac{{I}_{t}-{I}_{BGt}}{{I}_{pre}-{I}_{BGpre}}$$

where I_norm_ is the normalised FA intensity, I_t_ is the raw intensity of the FA at time point t and I_pre_ is the average raw intensity of the FA during the entire prebleach period, I_BGt_ and I_BGpre_ are the corresponding intensities of the average background signal for the experiment.

To facilitate comparison of the recovery rates for the different proteins irrespective of their final recovery levels the data was also normalised in such a way as to set the first value after bleaching to zero and the final recovery level to 1 using the following formula:

$${I}_{norm}=\frac{({I}_{t}-{I}_{BGt})-({I}_{0}-{I}_{BGo})}{({I}_{post}-{I}_{BGpost})-({I}_{0}-{I}_{BGo})}$$

where I_norm_ is the normalised FA intensity, I_t_ is the raw intensity of the FA at time point t, I_0_ at the first time point after bleaching and I_post_ the average raw intensity of the FA during the last 25 time points of the experiment, I_BGt_, I_BG0_ and I_BGpost_ are the corresponding average background signals for the experiment.

#### Fitting of the experimentally derived FRAP curves using Monte-Carlo based simulations

For analysis of FRAP data, FRAP curves were normalized to prebleach values. A database of Monte Carlo based computer simulated FRAP curves was generated in which four parameters representing mobility properties were varied: long and medium immobile fractions (random values between 0 and 70%) and time spent in immobile state, ranging from medium residence times (random values between 20 and 100 s) to long residence times (random values between 600 and 3200 s). Database sizes of 5122/2027 simulates FRAP curves were used for the analysis of the FRAP data from the U2OS or MDCK cells respectively. The simulated curves are based on a model of diffusion in an ellipsoid volume representing the cell with ellipsoid volumes representing FAs and simple binding kinetics representing binding to the FA complex. Simulations were performed at unit time steps of 100 ms. Results of the simulation were evaluated every 500 ms corresponding to the experimental sample rate. The diffusion coefficient of 1 μm^2^/s was based on separate experiments measuring free diffusion of paxillin-GFP in the cytoplasm. Diffusion was simulated by deriving novel positions (*x*_*t+*Δ*t*_, *y*_*t+*Δ*t*_, *z*_*t+*Δ*t*_) at each time step *t + *Δ*t* for all mobile molecules from their current positions (*x*_*t*_, *y*_*t*_, *z*_*t*_) by *x*_*t+*Δ*t*_ = *x*_*t*_ + G(*r*_1_), *y*_*t+*Δ*t*_ = *y*_*t*_ + G(*r*_2_), and *z*_*t+*Δ*t*_ = *z*_*t*_ + G(*r*_3_), where *r*_*i*_ is a random number (0 ≤ *r*_*i*_ ≤ 1) chosen from a uniform distribution, and G(*r*_*i*_) is an inverse cumulative Gaussian distribution with μ = 0 and *σ*^2^ = 2*Dt*, where *D* is the diffusion coefficient and *t* is time measured in unit time steps.

Immobilisation in FAs was based on simple binding kinetics with two immobile fractions, a medium and a long fraction:

$${M}_{mob}\leftrightharpoons {M}_{imm,medium}$$

$${M}_{imm,medium}\to {M}_{imm,long}\to {M}_{mob}$$

where M_mob_ are the mobile molecules and M_imm,medium_ and M_imm,long_ are the molecules in the medium and the long immobile fractions respectively.

Each mobile molecule in the simulation can bind at the adhesion for a medium length of time with a given chance. Once a molecule becomes a part of the medium immobile fraction it has a chance to either become mobile again or to become a part of the long immobile fraction. Molecules in the long immobile fraction have a chance of becoming mobile again. These chances are defined in accordance with the following kinetics described by:

$$koff=\frac{1}{{t}_{r}}$$

where *k*_*off*_ is the off rate constant in s^−1^ for the medium or the long immobile fraction and *t*_*r*_ is the average time in s spent immobile for molecules in this fraction.

$${k}_{on,medium}=\frac{{F}_{imm,medium}}{1-{F}_{imm,medium}-{F}_{imm,long}}\cdot ({k}_{off,medium}+{k}_{on,long})$$

where *k*_*on*,*medium*_ and *k*_*on*,*long*_ are the effective on rate constants in s^−1^ for the medium/long immobile fractions and *F*_*imm*,*medium*_*/F*_*imm*,*long*_ are the relative number of medium/long immobile molecules respectively.

$${k}_{on,long}=\frac{{F}_{imm,long}}{{F}_{imm,medium}}\cdot {k}_{off,long}$$

The ellipsoid volume of the cell was based on experimentally derived estimates of cell size, for U2OS cells this corresponds to a width of 24 µm, a length of 44 µm and a height of 2 µm, for MDCK to 15, 64 and 2 µm respectively. In each cell 2 ellipsoid volumes with widths of 1.5 µm, lengths of 2 µm and heights of 0.5 µm were used to simulate FAs.

The FRAP procedure was simulated on the basis of an experimentally derived 3D laser intensity profile providing a chance for each molecule to be bleached, based on its 3D position, during simulation of the bleach pulse. The number of fluorescent molecules in the ellipsoid volume of the bleached adhesion was used as the output of the simulation.

The experimentally derived FRAP curve for each individual FA was individually fitted to the simulated FRAP curves and the best fitting (least squares) curve was determined. For the parameters of interest, *k*_*on*,*medium*_ and *k*_*off*,*medium*_ we determined the interquartile range (IQR). Any FAs for which the best fitting curve resulted in a *k*_*on*,*medium*_ or *k*_*off*,*medium*_ outside of 1.5*IQR, the next best fitting curve was used iteratively until the parameter fell within 1.5*IQR range.

To give the resultant kinetic parameters for a set of FAs of interest the average was taken of the parameters corresponding to the best fitting curves for these FAs.

### Photoconversion experiments

#### Imaging

All photoconversion data was acquired on a Zeiss Elyra PS1 system equipped with a LSM 780 confocal unit set to confocal mode and using a 63× 1.4NA or a 100× 1.49NA oil immersion objective. For excitation a 561 nm/488 nm laser was used, the emission filters were set to 578–665 nm/490–525 nm for the green/cyan channel respectively, with a pinhole size of 59 µm and line averaging set to 4. For each experiment 2 images were taken of the green and red channel (t_pre_), followed by the photoconversion of 1–2 FAs by exposing a small rectangle drawn tightly around the FA(s) to 25 iterations of low intensity 405 nm laser light using the FRAP mode of the Zen software, immediately followed by the acquisition of another 2 images for both channels (t_0_) and the starting of a stopwatch. When the stopwatch showed 3 minutes had passed another 2 images of both channels were acquired (t_post_). For U2OS cells expressing paxillin-mMaple3 189 FAs were photoconverted from 153 cells, for U2OS cells expressing vinculin-mMaple3 98 FAs from 84 cells.

#### Data analysis

Data was analysed in the ImageJ software package in the Fiji framework, using a series of home-made macros. Firstly we made ‘ratio view’ (RV) images, where a fire-LUT was used to show on a pixel by pixel basis the ratio of the average intensity of the red signal for the two images taken at t_post_ over the average red signal for the two images taken at t_0_ (RVimage = I_avgredpost_/I_avgredt0_). The fire-LUT was only applied to pixels that at t_pre_ in the green channel had an above-threshold value (variable threshold depending on the intensity of the background cytoplasmic signal), reliably selecting FAs and a limited number of relatively high intensity background cytoplasmic pixels. Next we saved the FAs as ROIs using the Fiji build-in dynamic ‘moments’ threshold on an average projection of the green channel at t_pre_ and differentiated between high (stably bound) and low (dynamically bound) ratio pixels by applying this same dynamic threshold to the RV image. This visualised the stably and dynamically bound fractions of the protein separately. To determine whether the stably bound fractions of paxillin and/or vinculin concentrated into specific areas of the FA, we used Fiji’s build-in ‘analyse particles’ on the thresholded RV images to group together any above threshold pixels together covering a surface area of at least 0.05 µm^2^ and saved these as ROIs. For further analysis these ROIs were read into R using the Rstudio (R development Core Team, 2016), with custom-written R-scripts we plotted for each FA the ROI(s) corresponding to the stably bound area(s) in (shades of) red and the ROI corresponding to the rest of the FA in green, for easy comparison between FAs these plots are rotated relative to a virtual membrane to the left of these plots based on the orientation of the FA relative to the closest ventral membrane edge in the cell.

### classification of FAs

To examine the effects of FA location and/or orientation all FAs were classified. FAs were classified as ‘outer’ when they were located close to the ventral (adherent) membrane edge. FAs were classified as ‘inner’ when they were located further inwards, with another FA located between them and the closest membrane edge. FAs were classified as ‘pointing’ when they were orientated with their long axis ‘perpendicular’ (*i*.*e*. 90° ± 30°) to the closest ventral membrane edge. FAs were classified as ‘parallel’ when they were orientated with their long axis ‘parallel’ (*i*.*e*. 180° ± 30°) to the closest membrane edge. FAs outside these boundaries were discarded in these analyses, which for the FRAP data amounted to 31 of 233 FA for VASP (13%), 56 of 464 FAs for zyxin (12%), 62 of 332 FAs for paxillin (19%) and 35 of 249 FAs for vinculin (14%). Overall, from the FRAP data 184 of the 1278 bleached FAs (14%) fell outside the criteria for ‘pointing’ and ‘parallel’ and were discarded from analyses looking at FA orientation. Of the FAs selected for the photoconversion experiments for paxillin 13 of 189 FAs (7%) and for vinculin 1 of 98 FAs (1%) were discarded from analyses involving FA orientation for these reasons.

### Statistical analysis

For analyses of the differences between two groups two-tailed Mann-Whitney *U* tests were used. For analyses of the differences between more than two groups two-tailed Kruskall-Wallis Rank Sum tests were used, if this generated a p-value < 0.05 the specific groups with significant differences were determined using two-tailed Mann-Whitney *U* tests. To curtail the number of Mann Whitney *U* tests to be performed on the FRAP data separated based on FA location and orientation into four groups only the differences between meaningful combinations were analysed limiting the number of tests to 4 per parameter: outer pointing versus inner pointing, outer parallel versus inner parallel, outer pointing versus outer parallel and inner pointing versus inner parallel. As a further precaution against an inflated type I error rate, for all FRAP data where the FAs were separated on the basis of FA location and/or orientation the p-value was adjusted to < 0.01 to denote significance.

## Supplementary information

Supplemental Figures
