# Supplementary material for: Dynamics and distribution of paxillin, vinculin, zyxin and VASP depend on focal adhesion location and orientation
Source: Sci Rep. 2019 Jul 18;9:10460. doi: 10.1038/s41598-019-46905-2 (PMC6639384; doi:10.1038/s41598-019-46905-2)
Supplement: Supplementary file 1 — Supplemental Figures [file 41598_2019_46905_MOESM1_ESM.pdf]

Dynamics and distribution of paxillin, vinculin, zyxin and VASP depend on focal adhesion location and orientation

Karin Legerstee, Bart Geverts, Johan A. Slotman and Adriaan B. Houtsmuller

supplemental figures & legends

S 1

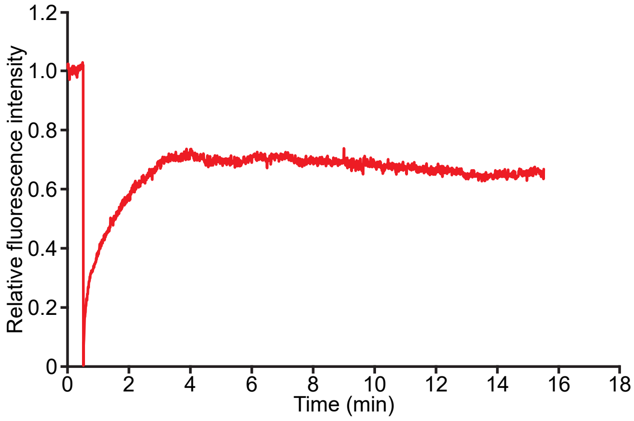

Supplementary Fig. S1 Prolonged FRAP applied to paxillin in MDCK cells

Fluorescence intensity of paxillin-GFP expressed relative to prebleach levels and intensity immediately after the bleach.

S 2

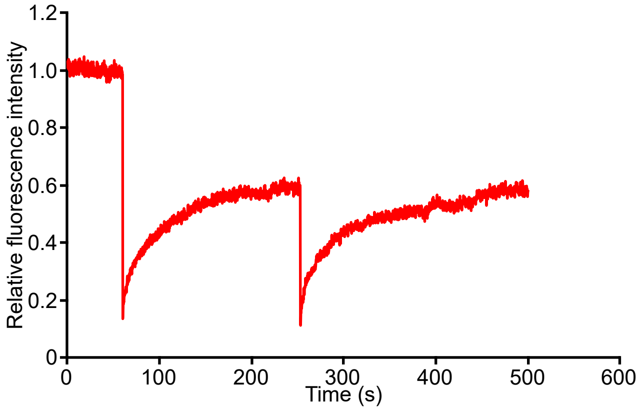

Supplementary Fig. S2 Repeated FRAP applied to paxillin in MDCK cells

Fluorescence intensity of paxillin-GFP expressed relative to prebleach levels. The bleach pulse was repeated after five mintutes

S 3

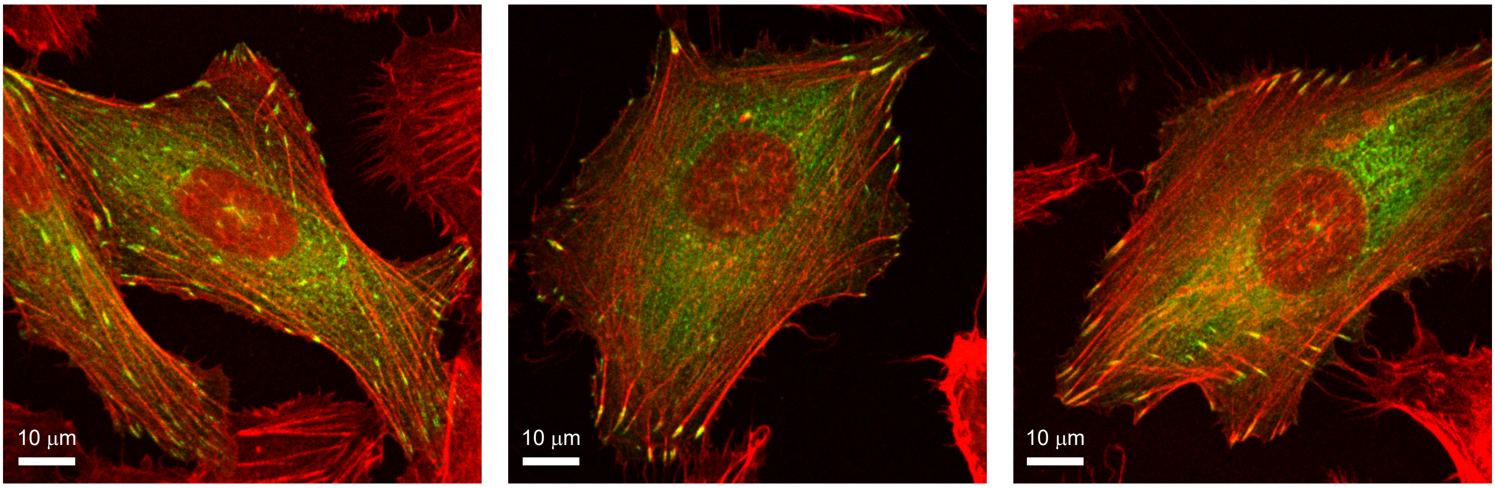

Supplementary Fig. S3 F-actin fibres enter pointing FAs at their proximal ends

Overlay images of U2OS cells stably expressing paxillin-GFP (green) stained with phalloiding-CF405 to highlight the F-actin (red)
